# Persistent Hypercalcemia Crisis and Recurrent Acute Pancreatitis Due to Multiple Ectopic Parathyroid Carcinomas: Case Report and Literature Review of Mediastinal Parathyroid Carcinoma

**DOI:** 10.3389/fendo.2020.00647

**Published:** 2020-09-10

**Authors:** Ruizhi Jiajue, An Song, Ou Wang, Wei Li

**Affiliations:** Key Laboratory of Endocrinology, Department of Endocrinology, National Commission of Health, Peking Union Medical College Hospital, Chinese Academy of Medical Science, Beijing, China

**Keywords:** primary hyperparathyroidism, ectopic parathyroid tumors, mediastinal parathyroid carcinoma, acute pancreatitis, hypercalcemia crisis

## Abstract

Mediastinal parathyroid carcinoma (PC) is a rare entity in primary hyperparathyroidism. The aim of this report is to demonstrate a case of mediastinal PC, and to provide a systemic literature review of this rare condition. A 34-year-old woman who had already undergone two cervical operations for hyperparathyroidism suffered from another recurrence, presenting with recurrent acute pancreatitis and persistent hypercalcemic crisis. Technetium-99 methoxyisobutylisonitrile imaging (MIBI) and computed tomography scanning (CT) identified three possible parathyroid tumors, one of which was the recurrence of residual tumor locating in the thyroid region, while the other two were ectopic tumors locating in the suprasternal fossa and thymus region, respectively. Pathological examination confirmed the diagnosis of PC. We conducted a systemic literature review by searching the PubMed MEDLINE from 1951 to 2019 for studies of all types in the English language only, using terms “mediastinal, mediastinum, parathyroid, carcinoma.” Including our reported case, a total of 21 cases with ectopic mediastinal PCs were assessed for demographic data, tumor location and size, biochemical findings, and symptomatology, etc. Two thirds of the patients were men, with a mean age of 44 years old, a mean serum calcium of 14.2 mg/dl, and a mean serum intact parathyroid hormone of 1,216 pg/ml. We identified 89.5% of carcinomas in the anterosuperior mediastinum, and 10.5% in the middle mediastinum, with a mean diameter of 54 mm, and a mean weight of 216 g. MIBI and CT were the most commonly used methods to localize these mediastinal tumors, with 69.2 and 100% sensitivity, respectively. Half of the patients underwent more than one operation. Diagnosis and treatment of mediastinal PCs represent a challenge. Early suspicion, appropriate preoperative localization studies, and the cooperation of endocrinologists and surgeons are crucial in the effective management.

## Introduction

Parathyroid carcinoma (PC) is a rare phenomenon. Following its original description in 1933, <300 cases are reported in the English literature (1), comprising from 1% (in the US and Europe) to 5% (in Japan and Italy) of all cases of primary hyperparathyroidism and only 0.005% of all tumors (2, 3). Although most cases normally occur in the cervical parathyroid glands, 6–16% of parathyroid tumors can be found outside of the cervical region, most often in the thyroid (18%), the thymus (38%), or behind the esophagus (31%) (4).

Primary PC is rarely detected in the mediastinum, only about 30 cases have been reported in the English literature so far (5–32). Its incidence in primary hyperparathyroidism varies from 0 to 11.8% in some case series (33–35). Until now, our knowledge of this special entity is still limited, and the diagnosis and treatment remain problematic and controversial.

The aim of this study is to present the diagnosis and management procedures in a complicated case with persistent hypercalcemia crisis and recurrent acute pancreatitis due to multiple ectopic parathyroid carcinomas, including a giant carcinoma in the anterosuperior mediastinum, and to conduct the largest literature review of mediastinal parathyroid carcinoma.

## Case Presentation

**Figure 1:**
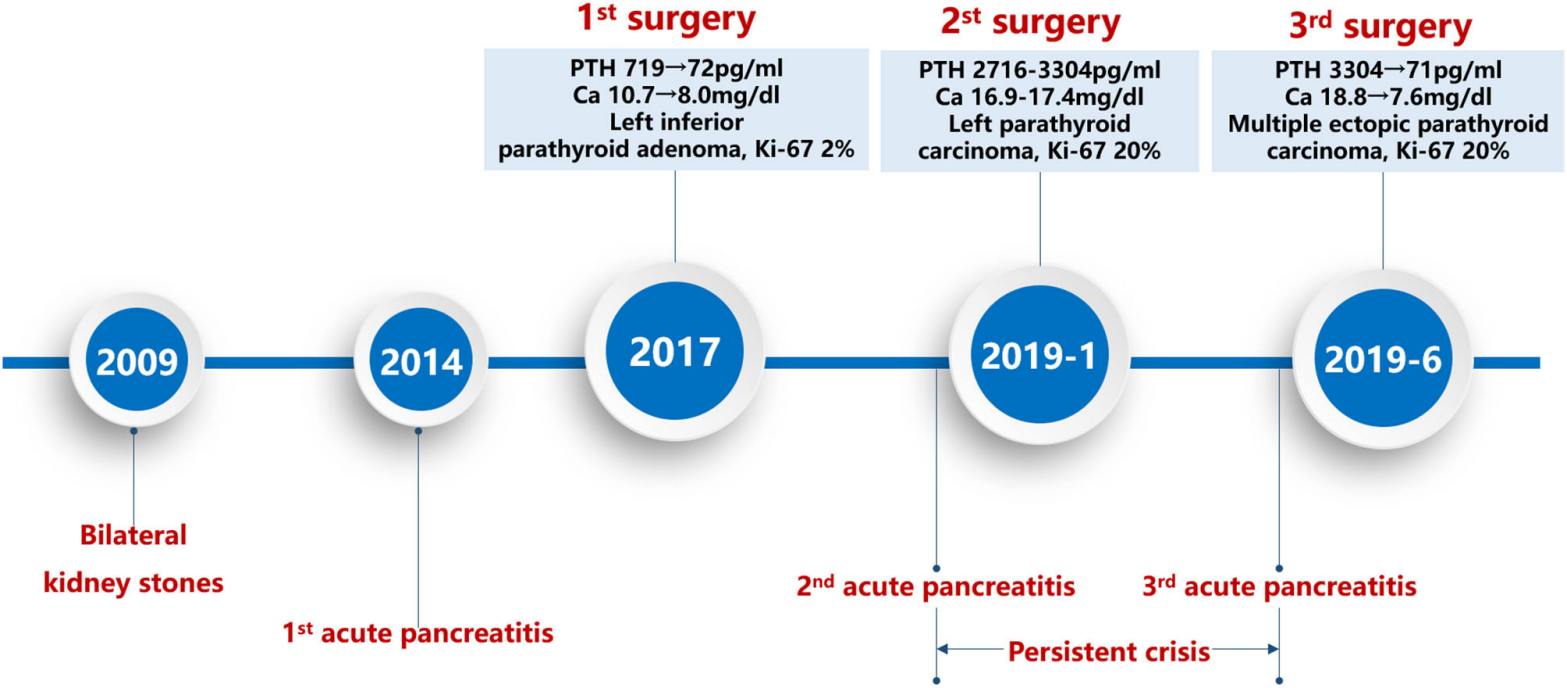
Event timeline. PTH, intact parathyroid hormone; Ca, calcium.

In 2017, a 34-year-old Chinese woman with a history of bilateral kidney stones, osteitis fibrosa cystica, and acute pancreatitis was diagnosed with primary hyperparathyroidism (Figure 1). Her serum level of intact parathyroid hormone (PTH) was 719 pg/ml (normal reference 12–68 pg/ml) and serum calcium was 10.7 mg/dl (normal reference 8.8–10.4 mg/dl). Left inferior parathyroid gland was resected, as the preoperative ultrasound indicated abnormal hyperplasia. Pathological examination confirmed the diagnosis of a parathyroid adenoma (PA) (2.0 × 1.5 cm, Ki-67 2%). Her serum levels of PTH and calcium decreased promptly several days after the surgery (PTH 72 pg/ml, calcium 8.0 mg/dl).

**Figure 2:**
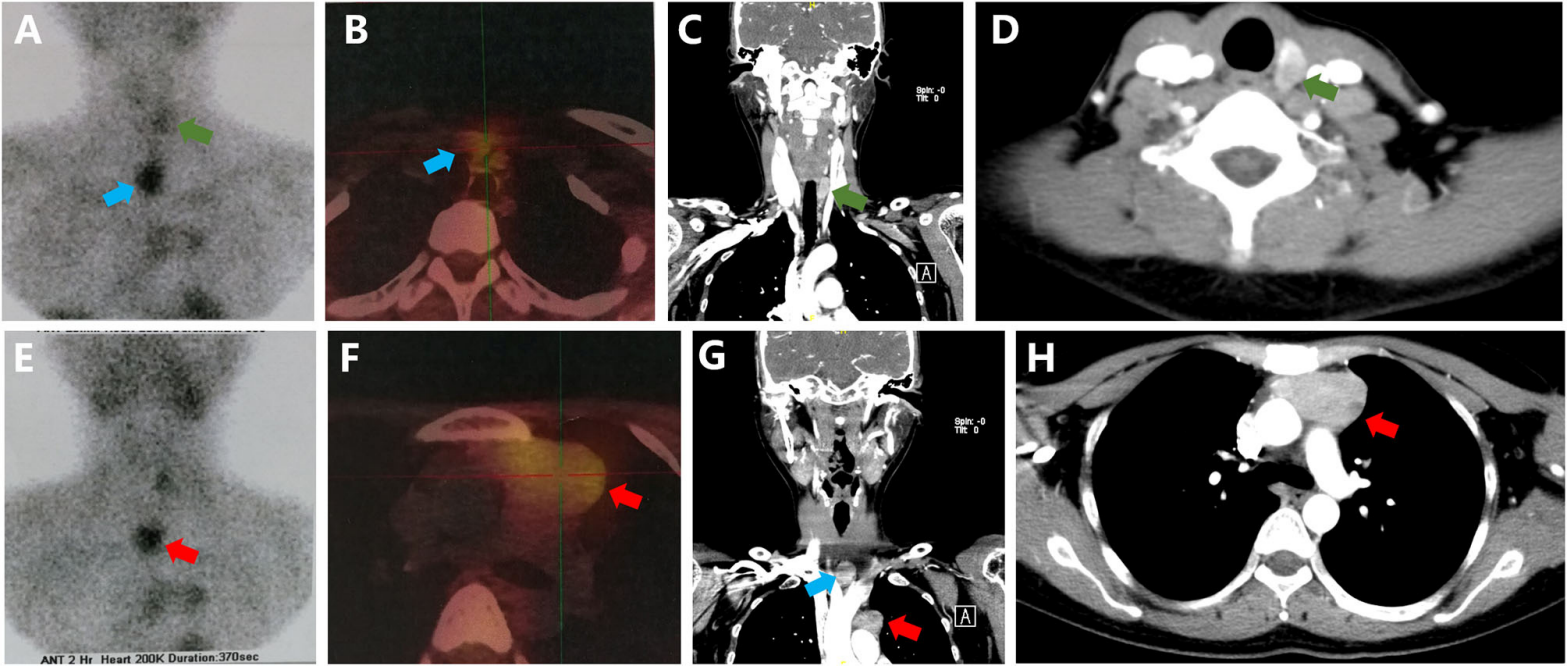
Imaging findings. **(A,B,E,F)** Technetium-99 methoxyisobutylisonitrile (99mTc-MIBI) imaging; **(C,D,G,H)** Enhanced computed tomography scanning. Green arrow for the parathyroid carcinoma in left inferior thyroid region, blue arrow for the carcinoma in the suprasternal fossa, and red arrow for the carcinoma in the thymic region.

In January 2019, she suffered from another acute pancreatitis, and the biochemical evaluation confirmed the recurrence of primary hyperparathyroidism (serum PTH 2,716–3,304 pg/ml and calcium 16.9–17.4 mg/dl). Due to the hypercalcemic crisis, emergency surgery was performed. Multiple nodules were detected in bilateral thyroid and a probable tumor was identified in the left inferior parathyroid gland. Subtotal excision of the bilateral thyroid and excision of the parathyroid tumor were performed. Pathological examination showed a parathyroid carcinoma (2 × 1 cm, Ki-67 20%) and a papillary carcinoma in the right thyroid gland. However, hypercalcemic crisis continued to deteriorate (Ca 18.8 mg/dl) and she experienced the third attack of acute pancreatitis in Jun 2019. Ectopic tumors were speculated and different imaging examinations were performed, such as parathyroid ultrasound, Technetium-99 methoxyisobutylisonitrile (99mTc-MIBI) imaging, and parathyroid enhanced computed tomography scanning. Beyond our speculation, multiple tumors were detected, including one in the left inferior thyroid region, which we believed to be the recurrence of the residual tumor, and two ectopic tumors, locating in the suprasternal fossa and in the thymus region, respectively (Figure 2). There was no evidence of multiple endocrine neoplasia syndrome (MEN), and genetic analysis of *CDC73/HRPT2* was negative. Her bone mineral densities measured using dual-energy X-ray absorptiometry (Lunar DPX; Lunar Corporation, Madison, WI, USA) was markedly decreased in the femoral neck (0.478 g/cm^2^, Z = −3.1), total hip (0.483 g/cm^2^, Z = −3.4), and lumbar spine (0.707 g/cm^2^, Z = −2.6). She did not report any clinical fractures. We also performed radiographic images of vertebrae, pelvis, and extremities to reveal occult fractures, and the results were negative. Her past medical history was unremarkable. No family member had a medical record of hyperparathyroidism, hypercalcemia, renal stones, osteoporosis, and fractures.

Combined medical treatments were prescribed first to control the hypercalcemic crisis, such as 4–5 L of saline by intravenous infusion every day, 20–40 mg furosemide by intravenous injection every day, 200 IU salmon calcitonin by intramuscular injection every 6 h, 4 mg zoledronic acid by intravenous infusion every 2 weeks (a total of three dosages were given in about one and a half months), and 50 mg cinacalcet taking orally every day. Although the patient felt much better and her abdominal pain gradually relieved with these treatments, she did not tolerate the cinacalcet well and experienced moderate nausea and vomiting. Moreover, she seemed to be refractory to these medical treatments with her serum calcium (12.0–12.8 mg/dl) remained higher than the normal range. We comprehensively explained to the patient about the risks and benefits of different therapeutic strategies, such as a long-term medical therapy and reoperation. The patient was more concerned about the side effects of the drugs and the metastasis of the tumors. Therefore, she strongly demanded for the third surgery. Transcervical surgery was conducted to resect the tumor in the thyroid region, and median sternotomy was performed to resect tumors in the suprasternal fossa and thymus region. Pathological examinations confirmed the diagnosis of parathyroid carcinomas. These tumors were round or oval shape surrounded by thick fibrous grayish capsule. Tumor size was 20 × 10 × 5 mm in the thyroid region, 35 × 20 × 10 mm in the suprasternal fossa, and 60 × 35 × 30 mm in the thymic region. The slice surface had a lobulated and whitish-gray appearance. Carcinoma infiltrations were detected in the thyroid tissue, thymic tissue, and fibrous tissue, as well as a high mitotic activity. Immunochemical examinations showed PTH (+), Calcitonin (±), PAX-8 (+), Syn (+), P53 (−), cyclin D1 (+), CEA (−), CgA (+), and Ki-67 20%. Her serum levels of PTH (71–108 pg/ml) and calcium (6.4–6.8 mg/dl) significantly decreased postoperatively. She felt some numbness and weakness in her limbs without tetany, and the Chvostek's sign was positive. Hungry bone syndrome was considered, and she was treated with large oral doses of calcium carbonate (3 g/day) and calcitriol (1 ug/day). Her discomfort gradually relieved and her serum calcium remained between 7.6 and 8 mg/dl. The patient was subsequently well without evidence of recurrence during a 6 months follow-up.

This patient was evaluated at the Peking Union Medical College Hospital (PUMCH). This report was approved by the Department of Scientific Research, the ethics committee in PUMCH. Informed consent form was signed by the patient, for the publication of clinical data.

## Methods

A systemic literature review was performed by searching the PubMed MEDLINE from March 1951 to August 2019 for studies of all types reporting the mediastinal parathyroid carcinoma in human, using the terms “mediastinal, mediastinum, parathyroid, carcinoma.” The search returned 613 publications in the English language only. A total of 586 publications were excluded as not relevant to the search criteria (227 publications focused on the parathyroid adenoma only; 137 investigated localization methods; 78 reported other parathyroid diseases or mediastinal diseases, such as the parathyroid carcinoma without mediastinum involvement, mediastinal parathyroid diseases without pathological confirmation, parathyroid hyperplasia, lung cancer, and lymphoma; 67 focused on the treatment of parathyroid tumors; 8 described clinical features of hyperparathyroidism and 69 were not inherent to our research).

Among the remaining 27 publications, 6 publications with 14 cases were further excluded due to an ambiguous diagnosis or the extremely incomplete information. We found 7 other articles by manually searching references of retrieved articles. The full texts of these 28 articles with 30 cases were assessed for eligibility. We summarized the detailed information of these 31 cases (including our case) in Table 1, categorizing by demographic data, tumor location and size, biochemical findings, symptomatology, and prognosis.

**Table 1:** Literature review of mediastinal parathyroid carcinomas.

**A/G**	**Location/diameter (mm)/Weight (g)^***a***^**	**Operation Num (n/N)^***b***^**	**Pre → Post- Ca (mg/dl)^***c***^**	**Pre → Post- PTH (pg/ml)^***c***^**	**Prognosis**	**Remarks (Symptoms, other parathyroid abnormalities, etc.)**	**References**
**Ectopic mediastinal parathyroid carcinomas**							
34/F	Thymic/60/NA	3/3	18.8–6.4	3,304 → 71	No relapse in 6 months	Recurrent pancreatitis, kidney stones, OFC. 1 cervical parathyroid adenoma, 2 carcinomas in thyroid region and suprasternal fossa	Our case
72/M	Ant-Sup/20/1.8	1/1	14.0 → 9.4	168 → 45	No relapse in 3 years	Acute pancreatitis, kidney stones. No other parathyroid tumor	(6)
54/M	Ant-Sup/40/NA	NA	24.8 → NA	NA	Death from cardiac shock	Renal failure. Postmortem confirmed carcinoma. No other parathyroid tumor	(8)
28/M	Superior/42/NA	1/1	23.3 → 8.2	2,480 → 21^d^	No relapse in 1 year	Obstruction of superior vena cava. No other parathyroid tumor	(10)
65/F	Superior/NA/NA	1/1	8.8 → NA	37 → NA	No relapse in 4 years	Non-functioning carcinoma. No other parathyroid tumor	(11)
23/M	Anterior/36/14.1	1/1	14.3 → 9.2	2,480 → 11^d^	No relapse in 2 years	Recurrent fractures, OFC, osteoporosis. No other parathyroid tumor	(12)
50/M	Superior/20.6/NA	2/2	10.6 → 9.7	415 → 8.2	Lost to follow-up	Giant neck parathyroid mass with mediastinal extension	(5)
10/M	NA/NA/NA	1/2	15.5 → 6.5	300 → 3.3	No relapse in 18 months	Neck mass. No other parathyroid tumor	(13)
61/M	Superior/85/56.8	1/1	7.6 → 4.0	1,220 → 59	No relapse in 3 years	Kidney stones, neck mass. No other parathyroid tumor	(14)
84/F	Middle/89/NA	NA	11.2 → NA	230 → NA	NA	Progressive dyspnea, osetoporosis. Biopsy confirmed carcinoma. Unresectable due to its infiltration. No other parathyroid tumor	(15)
31/M	NA/28/NA	NA	9.7 → NA	2,475 → NA	NA	Four hyperplastic parathyroid glands were resected due to secondary HPT. A mediastinal carcinoma was identified on the recurrence	(16)
35/M	Ant-Sup/NA/NA	1/2	13.4 → NA	707 → NA	Death from relapse after 5 years	MEN1 (pancreatic gastrinoma with PTHrP positive staining). Mediastinal carcinoma was resected first, and three hyperplastic parathyroid glands were removed on the recurrence	(17)
62/F	Anterior/30/NA	2/2	14.5 → NA	296 → NA	No relapse in 2 years	Non-specific symptoms. No other parathyroid tumor	(18)
37/M	Ant-Sup/120/1200	1/1	14.4 → 8.4	NA	No relapse in 4 years	Giant neck mass. No other parathyroid tumor	(19)
57/F	Anterior/53/16.7	2/2	16.3 → 6.2	3,000 → NA	No relapse in 7 years	No other parathyroid tumor	(23)
69/F	Ant-Sup/140/NA	NA	9.2 → NA	319 → NA	Survival 4 years	Non-functioning tumor. Pleural effusion. Chemotherapy sensitive	(25)
10/M	Anterior/20/NA	2/2	15 → NA^d^	NA	No relapse in 3 months	No other parathyroid tumor	(26)
27/F	Superior/NA/NA	3/3	11.8 → 9.9	1,518 → 53	No relapse in 9 months	Renal failure. Four hyperplastic parathyroid glands were resected first due to tertiary HPT. On the recurrence, a low-grade parathyroid carcinoma was found in right lower thyroid lobe extending to the head of thymus, without abnormal lymph nodes	(27)
33/M	Superior/30/6.27	1/1	13.4 → 9.5	722 → 30	No relapse in 2 months	Non-specific symptoms. No other parathyroid tumor	(28)
43/M	Thymic/NA/NA	1/2	14.1 → NA	NA	Relapse after 7 years	Non-specific symptoms. Familial cases. Cervical and thymic parathyroid carcinomas were removed simultaneously. Another cervical adenoma and mediastinal tumor were found upon recurrence	(29)
44/M	Ant-Sup/50/NA	1/1	16.6 → 9.9	1,004 → 38	No relapse in 14 months	Kidney stones. Cystic carcinoma. No other parathyroid tumor	(30)
**Mediastinal parathyroid tumors without detailed pathological information to confirm the diagnosis of carcinoma**							
72/F	Thymic/30/NA	2/2	13.0 → 8.4^e^	808 → 6	No relapse in 1 year	Progressive ostealgia, osteoporosis. No other parathyroid tumor	(9)
47/M	Ant-Sup/NA/NA	1/1	17.5 → 8.6	6,500 → 170	No relapse in 9 months	Neck mass. No other parathyroid tumor	(21)
62/M	Anterior/NA/NA	3/3	13.6 → NA	NA	NA	Brown tumors, renal calculi. Cervical and mediastinal parathyroid carcinomas were revealed simultaneously. Angiography showed that the mediastinal tumor was supplied by the internal mammary artery	(31)
44/F	Thymic/13/NA	2/2	13.3 → NA	NA	NA	Kidney stones. Parathyroid hyperplasia. Cystic mediastinal carcinoma	(32)
**Metastatic mediastinal parathyroid carcinoma**							
49/M	Ant-Sup/30/NA	4/4	17.7 → 13.7	5,570 → 4,090	Death after fracture	Recurrent fractures, osteoporosis, nephrocalcinosis. Cervical parathyroid tumors (1 adenoma and 2 carcinomas). Bone metastases	(7)
45/M	Superior/NA/8	6/6	NA	NA	No relapse in 1 year	Cervical parathyroid carcinoma with metastasis in the lung and mediastinal lymph nodes	(20)
46/M	Superior/NA/5.6	2/2	17.6 → NA	NA	NA	Neck mass, OFC. Right upper parathyroid carcinoma. Metastatic lymph nodes in right jugular chain and superior mediastinum	(22)
33/M	Superior/30/NA	NA/8	18 → NA	NA	Death from VF 15 years after diagnosis	Pancreatitis, OFC. Postmortem confirmed metastatic mediastinal carcinoma. Cervical parathyroid tumors (adenoma and carcinoma)	(24)
55/F	NA	3/3	15.8 → NA	NA	Survival nearly 5 years	OFC. Cervical parathyroid tumors (adenoma and carcinoma) with metastatic lymph nodes in neck and mediastinum	(24)
49/M	NA	NA	NA	NA	Survival 9 months	Non-functioning tumor. Chemotherapy insensitive. Cervical carcinoma with metastasis in lung, bone and mediastinum	(24)

a *Location, maximum diameter, and weight of the mediastinal parathyroid carcinoma. Location is directly described in the literature or speculated from the radiological imaging*.

b *n = the number of the operation which resected the mediastinal carcinoma, N = the total operation numbers that the patient took*.

c *Pre → Post- = the serum levels of calcium and PTH before and after the treatment (operation or chemotherapy). Intact PTH value in some literatures (10, 12, 18, 27, 30) was reported in pmol/L unit, and was changed into pg/ml unit based on the formula (pmol × 9.43 = pg/ml), according to the Matejek et al. (36). C-terminal PTH measured in some literatures (19, 29) was not recorded in this table*.

d *Pre-operation PTH and calcium value was over the upper limit of detection, and was reported as the upper limit in this table*.

e *Only ionized calcium was reported in this literature. Total calcium value was estimated based on the formula (Total calcium = ionized calcium × 2)*.

*A/G, age (years)/Gender; M, male; F, female; Ant-Sup, anterosuperior; NA, not applicable; Ca, calcium; PTH, parathyroid hormone; OFC, osteitis fibrosa cystica; HPT, hyperparathyroidism; PTHrP, parathyroid hormone-related protein; VF, ventricular fibrillation*.

The diagnosis of parathyroid carcinoma is very complex. Sometimes, atypical PA can be easily misdiagnosed as PC in unexperienced hands (37, 38). Therefore, in order to guarantee the diagnosis accuracy of PC in prior cases, we conducted a further screening according to the following criteria: (1) a clinical diagnosis of PC based on metastases; (2) a pathological diagnosis of PC based on capsular, vascular, and/or perineural tumor invasion. There were 4 cases without the detailed pathological information to confirm the diagnosis of carcinoma. We further distinguished cases with more possibility to be a ectopic mediastinal PC rather than a mediastinal metastasis based on the following principles: (1) the mediastinal carcinoma was the only tumor being found; (2) the mediastinal carcinoma was found after four benign hyperplastic parathyroid glands were removed due to the secondary or tertiary hyperparathyroidism; (3) Angiography showed that the mediastinal tumor was supplied by the internal mammary artery, which suggests that its location is congenital resulting from developmental descent into the chest with the thymus gland; (4) No metastases were found. Six cases were believed to be metastatic lymph nodes in the mediastinum, and the other 21 cases (including our case) were reported as ectopic mediastinal PC. We analyzed and summarized the clinical, biochemical, radiological, and pathological characteristics of these 21 cases with ectopic mediastinal PC in Table 2.

**Table 2:** Summary of 21 cases with ectopic mediastinal parathyroid carcinomas.

**Parameter^**a**^**	**Mediastinal PC from literature (*n* = 21)**	**Ectopic PA from literature^**d**^**	**Mediastinal PA from PUMCH (*n* = 8)**	**Orthotopic PC from PUMCH (*n* = 20)**
Male: Female	2: 1	13: 17 (35) ~ 17: 21 (39)	1: 1	3: 2
Age (years)	44	44 (35) ~ 51 (39)	49	42
**Mediastinal location**				
Anterosuperior	89.5% (17/19)	92.1% (35/38) (39)	100% (8/8)	NA
Middle	10.5% (2/19)	5.3% (2/38) (39)	0	NA
Posterior	0	2.6% (1/38) (39)	0	NA
Diameter (mm)	54	24 (35)	30	26
Weight (g)	216.0	4.9 (35)	NA	4.0
≥2 Operation times	50.0% (9/18)	30% (3/10) (27)	25% (2/8)	40% (8/20)
Calcium (mg/dl)^c^	14.2	11.2 (40) ~ 12.6 (41)	12.2	14.2
Intact parathyroid hormone (pg/ml)^c^	1216	122 (40) ~ 236.4 (41)	697	1,087
**Complications**^**b**^				
Skeletal involvement	14.3% (3/21)	23% (3/13) (41) ~ 26% (10/38) (39)	25% (2/8)	40% (8/20)
Renal involvement	28.6% (6/21)	46% (6/13) (41) ~ 74.0% (28/38) (39)	25% (2/8)	70% (14/20)
Pancreatitis	9.5% (2/21)	0 (41)	0	0
Hypercalcemic Crisis	57.1% (12/21)	10% (1/10) (27)	12.5% (1/8)	65% (13/20)
**Positive localization methods**				
Computed tomography scan	100% (13/13)	46.2% (6/13) (42)	66.7% (4/6)	90% (9/10)
Magnetic resonance imaging	100% (3/3)	NA	NA	NA
Nuclear medicine imaging^c^	69.2% (9/13)	81% (161/197) (41)	75% (6/8)	80% (16/20)

a *Results of continuous and categorial variable are, respectively, expressed as mean value and percentage*.

b *Non-functioning carcinoma is not considered in the analyses of calcium, parathyroid hormone, and complications. Skeletal involvements include osteoporosis, fractures, and osteitis fibrosa cystica. Renal involvements include renal failure, kidney stones, renal calculi, and nephrocalcinosis*.

c *Nuclear medicine imaging was performed in 13 cases: 12 cases by ^99m^Tc sestamibi scintigraphy, and 1 case by thallium scan*.

d *Results derive from case series investigating ectopic parathyroid adenomas, mainly in the mediastinum. References of these literature were marked as superscript lowercase numbers*.

*PC, parathyroid carcinoma; PA, parathyroid adenoma; PUMCH, Peking Union Medical College Hospital; NA, not applicable*.

In order to compare the different features between mediastinal PCs, mediastinal Pas, and orthotopic PCs, we retrieved medical records of patients with mediastinal PAs or orthotopic PCs, who were admitted to the endocrinology department of PUMCH and underwent operations in the past 20 years. We selected 8 patients with mediastinal PAs and 20 patients with orthotopic PCs, and conducted an analyzation for biochemical, radiological, and pathological features of these patients (Table 2). We also tried to search literatures reporting case series of mediastinal PAs. However, only studies investigating ectopic PAs (most in the mediastinum) were found, and each study described different findings of ectopic PAs. These findings of ectopic PAs were also summarized in Table 2.

## Results and Discussion

Although the diagnosis of PC should be considered in patients with markedly evaluated PTH levels and severe hypercalcemia (43), these laboratory criteria are non-specific, and clinical criteria of local invasion and/or metastases are usually required. It is even more challengeable to distinguish PC from atypical PA at histological examination, since atypical PA share some features with PC, such as a diffuse growth pattern, fibrous speta, high mitotic activity. Capsular, vascular, and/or perineural tumor invasion are usually reliable indicators of malignancy. Other histologic features that might be useful in improving diagnostic accuracy of PC include attachment of tumor cells to the wall of the vessel, thrombosis, tumor cell necrosis, increased or atypical mitotic figures, higher expression of Ki-67, lower expression of p27, and overexpression of p53 and cyclin D1. Loss of nuclear parafibromin has also been shown to be a highly sensitive and specific marker for the diagnosis of PC (37, 38, 43). The diagnosis of our case is based on the combined findings from her clinical and pathological features. She is a very young female unfortunately suffered from severe symptoms of hyperparathyroidism, such as persistent hypercalcemic crisis, acute pancreatitis, and osteitis fibrosa cystica, which are more common in PC than in PA (38). She has markedly increased levels of serum calcium (>3 mmol/L) and PTH (over 10-fold elevation of the normal range). Her pathological findings show that the tumor has a size over 3 cm, infiltrations into the surrounding tissue, and a high Ki-67 index. All these biochemical and pathological features are found to be more related with PC (37, 38).

It is even more difficult to confirm whether the mediastinal mass is a primary tumor or a metastasis. Some features of our case convince us that her mediastinal tumor is more likely to be an ectopic parathyroid tumor rather than a metastasis. First, PC is an indolent tumor with low potential for distant metastasis. The common metastatic regions of parathyroid carcinoma are cervical lymph nodes, lung, liver, and bone, while mediastinum is a rare metastatic area. All the cases with mediastinal metastasis that we find in the literature are reported to be metastatic lymph nodes. However, we did not find any abnormal lymph nodes in our case during the surgery or on the pathological investigation. Second, it is not unusual that hyperfunctioning parathyroid glands are found in non-typical areas especially when they are diseased, considering about the parathyroid embryology. Among these non-typical areas, thymus is the most common one. Besides, we think thymus is a rare metastatic area of cervical tumors in consideration of their metastatic pathways.

We reported a very rare and complicated case of parathyroid carcinoma, which can be illustrated by three distinctive features. First, a total of five parathyroid tumors were resected from this patient, with 1 orthotopic adenoma and 2 orthotopic carcinomas identified in the left inferior parathyroid gland, and 2 ectopic carcinomas resected from the suprasternal fossa and the anterosuperior mediastinum, respectively. It is known from our literature review that the ectopic mediastinal parathyroid carcinoma is seldomly reported (5–32). Second, throughout the whole course of the disease, the patient prominently manifested with recurrent acute pancreatitis, the incidence of which in hyperparathyroidism is only 1.47% (44), and there is only one report of acute pancreatitis associated with the mediastinal parathyroid carcinoma (6). The last but not the least, along with her recurrence, this patient suffered from persistent hypercalcemic crisis. Although we adopted an potent therapy strategy using all the available calcium-lowering drugs, the outcome was unsatisfactory. The insensitivity of our case to all the medicine should be caused by her multiple hyperfunctioning parathyroid carcinomas.

In addition, we conducted the first and the largest literature review of the mediastinal parathyroid carcinoma, and compared its different features with the ectopic parathyroid adenoma and the orthotopic parathyroid carcinoma. Since Weissman et al. reported what we believe to be the first case of mediastinal parathyroid carcinoma in 1957 (45), add up to 44 cases with mediastinal parathyroid carcinoma were found by our systemic literature search, among which 31 cases were described as ectopic carcinomas and the other 13 cases were more likely to be metastatic lymph nodes in mediastinum. We only summarized and analyzed cases with definite confirmation of ectopic mediastinal parathyroid carcinoma.

Although the incidence of ectopic parathyroid glands in the mediastinum of general population is ~6% (46), and the estimation of mediastinal parathyroid adenomas in all parathyroid adenomas is nearly 10–20% (33), the incidence of mediastinal parathyroid carcinomas remains unknown. The unusual occurrence of mediastinal parathyroid carcinomas can be best illustrated by some case series. In two studies investigating cases with mediastinal parathyroid tumors, only 1 carcinoma of 84 cases (1.2%) (33) and 2 carcinomas of 17 cases (11.8%) (34) were, respectively, reported. Moreover, in the largest series reported by Wang et al., of 1,200 patients surgically treated for hyperparathyroidism, none presented with a mediastinal parathyroid carcinoma (35).

The occurrence of ectopic mediastinal parathyroid tumors is not surprising, in the sense that both the inferior parathyroid glands and the thymus originate from the third pharyngeal branchial pouch. The parathyroid glands may migrate into the anterosuperior mediastinum, either within or outside the thymus, or migrate along the esophagus into the posterosuperior mediastinum (33, 46, 47). Six to sixteen percent of PAs can be found in non-typical places—most often in the thyroid (18%), the thymus (38%), or behind the esophagus (31%) (4, 40). However, ectopic PCs are rarely reported, and the exact incidence is unclear. Through our literature search, we find that mediastinum with 21 cases and intrathyroidal region with 8 cases (48) are the most common locations of ectopic PCs, while other locations are barely reported. Consistent with mediastinal PAs (33, 39), nearly 90% of mediastinal PCs were found in the anterosuperior mediastinum (Table 2). Two other cases were identified in the middle mediastinum. However, no PC was found in the posterior mediastinum.

Despite of the location, PCs occur equally in males and females (38). However, mediastinal PCs are twice as common in males as in females (Table 2). In contrary, ectopic PAs seem to be slightly more frequent in females than males (female: male ratio = 1.2–1.3) (35, 39). Currently, there is no external independent reference standard for the diagnosis of PC, and the localization of mediastinal tumors are problematic. Therefore, clinical and biochemical parameters may be helpful in the differential diagnosis. In our analysis, the average value of diameter (54 mm), weight (216 g), serum calcium (14.2 mg/dl), and serum parathyroid hormone (1,216 pg/ml), as well as the frequency of hypercalcemic crisis (57.1%) in mediastinal PCs are all markedly higher than that of ectopic PAs, reported either in our hospital or in the literature (49). These findings consistent with the results comparing orthotopic PCs and orthotopic PAs (38, 50). In the comparison between mediastinal PCs and orthotopic PCs, they have similar serum levels of calcium and parathyroid hormone, and a similar high risk of hypercalcemia crisis. However, the mediastinal PCs are much larger and heavier than the orthotopic PCs.

The most commonly affected organ systems in patients with primary hyperparathyroidism are the renal and skeletal systems (5). Renal involvements include nephrolithiasis, nephrocalcinosis, and impaired glomerular filtration, while skeletal abnormalities secondary to hyperparathyroidism are usually reported as osteoporosis, osteitis fibrosa cystica, fractures, and salt-and-pepper skull. The frequencies of skeletal and renal complications were similar between ectopic carcinomas and adenomas (Table 2), but lower than that of orthotopic carcinomas, while higher than that of benign parathyroid hyperparathyroidism (<20% renal impairment and <10% skeletal abnormalities) (5, 42). Although neither we nor Mendoza et al. (41) report acute pancreatitis in ectopic PAs, there are a few case reports describing mediastinal PAs with the presentation of acute pancreatitis (51, 52). Therefore, large-scale cohort studies are needed to investigate the association between pancreatitis and PCs.

In cases with mediastinal parathyroid tumors, the crucial stage is the localization of the malignant lesion, especially in terms of its size and invasion, in order to allow for a radical surgery. One of the simplest, safest, and repeatable imaging is ultrasonography. However, its sensitivity (50–60%) is insufficient and it can mainly be used in localizing lesions in the neck and thyroid (7). In our summary of mediastinal PCs, only 1 out of 11 (9.1%) cases performing ultrasonography successfully identified the mediastinal tumor mainly due to its giant size (3). An alternative test is ^99m^Tc sestamibi scintigraphy which is proven to be effective in localizing ectopic lesions and distant metastases (40, 53). We reported an accuracy of 66.7% (8/12) of sestamibi scintigraphy in localizing mediastinal PCs, which is mildly lower than that of mediastinal PAs (75%) and orthotopic PCs (80%) in our hospital, and that of Roy et al. (40) investigating ectopic PAs (81%). The accuracy of computed tomography scan (CT) and magnetic resonance (MRI) imaging in detecting PCs (CT 90–100% and MRI ~100%) are much higher than that in detecting PAs (CT 43–92% and MRI 50–93%) (39, 46). Better results can be obtained using hybrid imaging combining MIBI scintigraphy and CT or MRI (54). All eight cases adopting hybrid imaging (sestamibi scintigraphy + CT ± MRI) in our review successfully localized the mediastinal carcinomas.

Primary hyperparathyroidism is the main endocrinopathy associated with MEN1 (>90%) and is typically the first endocrine manifestation. Almost all the hyperparathyroidism associated with MEN1 are caused by multiple parathyroid adenomas or hyperplasia, the incidence of PC in MEN1 is only 0.28–1% (55, 56). Although rare, its presence is noteworthy because it affects the extent and complexity of surgical approach. Therefore, it is also necessary to investigate MEN1 for patients with PC.

There are some inevitable limitations of our study. Positive parafibromin immunohistochemical staining is important in the diagnosis of PC (53). Therefore, lack of parafibromin staining of our case might make our diagnosis of PC less persuasive. However, we think that our diagnosis is unequivocal based on the combined findings aforementioned. As to the literature review, we might still omit some related cases even with such a broad systemic literature search. Although we have carefully assessed all the articles retrieved, and select cases of ectopic mediastinal PCs under strict criteria, some misdiagnosed cases might also be included. Considering the limited number of patients in this study, our results need to be validated in a large-scale study.

In conclusion, we reported a rare case of parathyroid carcinoma, who had multiple ectopic carcinomas, including a giant one in the anterosuperior mediastinum, and presented with very severe complications of hyperparathyroidism, such as recurrent acute pancreatitis and persistent hypercalcemic crisis insensitive to many calcium-lowering agents. From the largest literature review of mediastinal parathyroid carcinomas we provided, it should be known that the extreme rarity of this condition pose a lot of diagnostic and therapeutic challenges for clinicians. Early suspicion of mediastinal parathyroid tumors in a patient, conducting appropriate preoperative localization studies, and the cooperation of experienced endocrine specialists and cervicothoracic surgeons are crucial in the effective management.

## Data Availability Statement

All datasets generated for this study are included in the article/supplementary material.

## Ethics Statement

The studies involving human participants were reviewed and approved by the Department of Scientific Research, the Ethics Committee in PUMCH. The patients/participants provided their written informed consent to participate in this study. Written informed consent was obtained from the individual(s) for the publication of any potentially identifiable images or data included in this article.

## Author Contributions

RJ and WL contributed to conceive, design, and wrote the review. AS and OW contributed to collect the data and revised the review. All authors read and approved the final manuscript.

## Conflict of Interest

The authors declare that the research was conducted in the absence of any commercial or financial relationships that could be construed as a potential conflict of interest.
